# Markers of Celiac Disease and Gluten Sensitivity in Children with Autism

**DOI:** 10.1371/journal.pone.0066155

**Published:** 2013-06-18

**Authors:** Nga M. Lau, Peter H. R. Green, Annette K. Taylor, Dan Hellberg, Mary Ajamian, Caroline Z. Tan, Barry E. Kosofsky, Joseph J. Higgins, Anjali M. Rajadhyaksha, Armin Alaedini

**Affiliations:** 1 Department of Medicine, Columbia University, New York, New York, United States of America; 2 Celiac Disease Center, Columbia University, New York, New York, United States of America; 3 Kimball Genetics, a Division of LabCorp, Denver, Colorado, United States of America; 4 Center for Clinical Research, Uppsala Univeristy, Falun, Sweden; 5 Department of Neurology & Neuroscience, Weill Cornell Medical College, New York, New York, United States of America; 6 Department of Pediatrics, Weill Cornell Medical College, New York, New York, United States of America; 7 Institute of Human Nutrition, Columbia University, New York, New York, United States of America; Centro di Riferimento Oncologico, IRCCS National Cancer Institute, Italy

## Abstract

**Objective:**

Gastrointestinal symptoms are a common feature in children with autism, drawing attention to a potential association with celiac disease or gluten sensitivity. However, studies to date regarding the immune response to gluten in autism and its association with celiac disease have been inconsistent. The aim of this study was to assess immune reactivity to gluten in pediatric patients diagnosed with autism according to strict criteria and to evaluate the potential link between autism and celiac disease.

**Methods:**

Study participants included children (with or without gastrointestinal symptoms) diagnosed with autism according to both the Autism Diagnostic Observation Schedule (ADOS) and the Autism Diagnostic Interview, Revised (ADI-R) (n = 37), their unaffected siblings (n = 27), and age-matched healthy controls (n = 76). Serum specimens were tested for antibodies to native gliadin, deamidated gliadin, and transglutaminase 2 (TG2). Affected children were genotyped for celiac disease associated HLA-DQ2 and -DQ8 alleles.

**Results:**

Children with autism had significantly higher levels of IgG antibody to gliadin compared with unrelated healthy controls (*p*<0.01). The IgG levels were also higher compared to the unaffected siblings, but did not reach statistical significance. The IgG anti-gliadin antibody response was significantly greater in the autistic children with gastrointestinal symptoms in comparison to those without them (*p*<0.01). There was no difference in IgA response to gliadin across groups. The levels of celiac disease-specific serologic markers, i.e., antibodies to deamidated gliadin and TG2, did not differ between patients and controls. An association between increased anti-gliadin antibody and presence of HLA-DQ2 and/or -DQ8 was not observed.

**Conclusions:**

A subset of children with autism displays increased immune reactivity to gluten, the mechanism of which appears to be distinct from that in celiac disease. The increased anti-gliadin antibody response and its association with GI symptoms points to a potential mechanism involving immunologic and/or intestinal permeability abnormalities in affected children.

## Introduction

Glutens are the major storage proteins of wheat and related cereals, comprising over 70 different molecules in any given wheat variety [1]. The main classes of gluten include α/β-gliadins, γ-gliadins, ω-gliadins, high molecular weight glutenins, and low molecular weight glutenins [2]. Gluten sensitivity can be defined as a state of heightened immunologic reaction to gluten proteins, which may be accompanied by increased levels of antibodies against them. Heightened immune reactivity to gluten is recognized and understood best in the context of celiac disease, an autoimmune disorder primarily targeting the small intestine, and wheat allergy [3]. The humoral immune response in celiac disease also includes antibodies to deamidated sequences of gliadin and to the autoantigen transglutaminase 2 (TG2), which are highly specific and sensitive serologic markers of the condition [4]. Celiac disease is also closely linked with genes that code for human leukocyte antigens (HLA) DQ2 and DQ8 [5].

While the etiology and pathogenesis of autism are poorly understood, there is evidence that immune system abnormalities are associated with symptoms in a substantial number of affected individuals [6]. In addition, several studies have evaluated gastrointestinal (GI) symptoms and defects in GI barrier function in patients with autism [7]–[10]. A possible association between autism and celiac disease was first discussed over 40 years ago [11], [12]. Although some studies have pointed to higher frequency of celiac disease, family history of celiac disease, or elevated antibody to gliadin among autistic children [13]–[15], others have not supported these findings [16]–[18]. Diets that exclude gluten are becoming increasingly popular in the autism community, but their effectiveness has not been proven in controlled and blinded studies [19]. Despite years of speculation and immense interest by families of affected children regarding the potential connection between autism and gluten sensitivity, no well-controlled study has been performed to determine the levels of immune reactivity to gluten in patients, to characterize the antigenic specificity of this immune response, or to assess its pathogenic relevance to autism. In this study, we examine and compare markers of celiac disease and gluten sensitivity in cohorts of individuals diagnosed with autism, unaffected siblings of the patients with autism, and unrelated healthy controls.

## Methods

### Patients and Controls

The study included 140 children, including 37 with autism, 27 unaffected siblings of similar ages within the same families, and 76 unrelated healthy controls. Serum samples from individuals with autism and their siblings were acquired from the Autism Genetic Resource Exchange (AGRE). DNA samples from the 37 children with autism were also provided by AGRE. Participants in the AGRE program have been recruited primarily from the northeastern and western United States. Affected children met the diagnostic criteria for autism based on both the Autism Diagnostic Observation Schedule (ADOS) and the Autism Diagnostic Interview, Revised (ADI-R). All available serum samples satisfying the above criteria were included. Information on GI symptoms was based on parent questionnaires, interviews, and medical histories. The data collected by AGRE from these evaluations were retrieved from the online AGRE phenotype database. The control sera were from healthy children in the United States (n = 14) and Sweden (n = 62). The healthy controls from U.S. resided primarily in Connecticut, north New Jersey, and New York City, and were recruited in a general pediatric clinic at the Weill Cornell Medical College. The healthy controls from Sweden were recruited at child health care centres and schools in the Falun region of central Sweden [20]. Screening questionnaires were used to evaluate the general health of the U.S. and Swedish controls, and individuals who reported having a chronic disease were not included. Serum from a biopsy-proven celiac disease patient, diagnosed according to previously described criteria [21] at Columbia University Medical Center, was used as a positive control for the antibody assays. Written informed consent was obtained for all study participants from the individual, next of kin, caretaker, or guardian. The consent procedures were approved by the Institutional Review Boards of the involved organizations (AGRE, Columbia University, Weill Cornell Medical College, and Uppsala University). Complete documentation of consent is maintained at the respective organizations. This specific study was approved by the Institutional Review Board of Columbia University Medical Center. Specimens were kept at −80°C to maintain stability.

### Gliadin

The antigen mixture used for the anti-gliadin antibody assays was the Prolamine Working Group (PWG) reference gliadin, which was extracted from a combination of 28 different wheat varieties, as previously described [22]. The protein profile of the PWG gliadin extract was assessed by SDS-polyacrylamide gel electrophoresis, using 10% NuPAGE Bis-Tris precast gels and 3-(N-morpholino)propanesulfonic acid (MOPS) buffer (Life Technologies, Carlsbad, Calif.).

### Anti-gliadin Antibodies

Serum IgG and IgA antibodies to gliadin were measured separately by enzyme-linked immunosorbent assay (ELISA) as previously described [23], [24], with some modifications. A 2 mg/mL stock solution of the PWG gliadin was prepared in 60% ethanol. 96-well Maxisorp round-bottom polystyrene plates (Nunc, Roskilde, Denmark) were coated with 50 µL/well of a 0.01 mg/mL solution of PWG gliadin in 0.1 M carbonate buffer (pH 9.6) or were left uncoated to serve as control wells. After incubation at 37°C for 1 h, all wells were washed and blocked by incubation with 1% bovine serum albumin (BSA) in phosphate buffered saline containing 0.05% Tween-20 (PBST) for 1.5 h at room temperature. Serum samples were diluted at 1∶800 for IgG measurement and at 1∶200 for IgA measurement, added at 50 µL/well in duplicates, and incubated for 1 h. Each plate contained a positive control sample from a patient with biopsy-proven celiac disease and elevated IgG and IgA antibodies to gliadin. After washing the wells, they were incubated with HRP-conjugated anti-human IgG (GE Healthcare, Piscataway, N.J.) or IgA (MP Biomedicals, Santa Ana, Calif.) secondary antibodies for 50 min. The plates were washed and 50 µL of developing solution, comprising of 27 mM citric acid, 50 mM Na_2_HPO_4_, 5.5 mM *o*-phenylenediamine, and 0.01% H_2_O_2_ (pH 5), was added to each well. After incubating the plates at room temperature for 20 min, absorbance was measured at 450 nm. All serum samples were tested in duplicate. Absorbance values were corrected for non-specific binding by subtraction of the mean absorbance of the associated BSA-coated wells. The corrected values were first normalized according to the mean value of the positive control duplicate on each plate. The mean antibody level for the unrelated healthy control cohort was then set as 1.0 AU and all other results were normalized accordingly.

### Anti-transglutaminase 2 (TG2) Antibodies

IgA antibody to recombinant human TG2 was measured in sera using an ELISA kit, according to the manufacturer’s protocol (Euroimmun, Lubeck, Germany).

### Anti-deamidated Gliadin Antibodies

Sera were tested separately for IgG and IgA antibodies to a previously described glutamine-glutamate substituted trimer of a fusion peptide containing the sequences PLQPEQPFP and PEQLPQFEE [25] by ELISA, according to the manufacturer’s protocols (Euroimmun).

### HLA Typing

High resolution HLA genotyping was performed by multiplex polymerase chain reaction (PCR) with biotinylated primers, followed by reverse hybridization of the PCR products to line arrays of sequence-specific DQA1 and DQB1 oligonucleotide probes, using INNO-LiPA HLA-DQ kits, according to the manufacturer’s instructions (Innogenetics, Gent, Belgium). Presence or absence of celiac disease-associated DQA1*0501/0505-DQB1*0201/0202 (DQ2) and DQA1*03-DQB1*0302 (DQ8) genes was determined.

### Data Analysis

Differences between groups were analyzed by the two-tailed Student’s *t* test, Welch’s *t* test, Mann-Whitney U test, or one-way analysis of variance (ANOVA) with post-hoc Dunn test (continuous data), and the Fisher’s exact test (nominal data). Adjustment for covariate effect (age, gender, and race) was carried out by analysis of covariance (ANCOVA), using the general linear model. Logistic regression was used to calculate the odds ratios associated with increased antibodies in individuals with autism. For these analyses, increased levels of anti-gliadin antibody were defined as values at the 95th percentile or higher in the unrelated healthy control group. For IgA anti-TG2 antibody and IgG/IgA anti-deamidated gliadin antibodies, cutoffs for positivity were assigned by the manufacturer. Differences with *p* values of *<*0.05 were considered to be statistically significant. Statistical analyses were performed with Prism 5 (GraphPad, San Diego, Calif.) and Minitab 16 (Minitab, State College, Pa.).

## Results

### Patients and Controls

The demographic and clinical characteristics of the patients with autism, their unaffected siblings, and unrelated healthy controls are shown in Table 1. The patient cohort included four individuals on gluten-free diet. Because the effect of gluten-free diet on antibody levels in autism is not known, these patients were not excluded from the study.

**Table 1:** Demographic characteristics of study cohorts.

Subject group		Number of subjects	Mean age– years ± SD	Male sex– no. (%)	White race no. (%)
**Autism**		**37**	**7.8±2.9**	**29 (78)**	**33 (89)**
	With GI symptoms	19	7.1±2.3	13 (68)	15 (79)
	Without GI symptoms	8	7.1±2.3	6 (75)	8 (100)
**Unaffected sibling**		**27**	**8.1±2.9**	**18 (67)**	**25 (93)**
**Unrelated healthy**		**76**	**8.8±3.7**	**59 (77)**	**70 (92)**

### Gliadin

**Figure 1:**
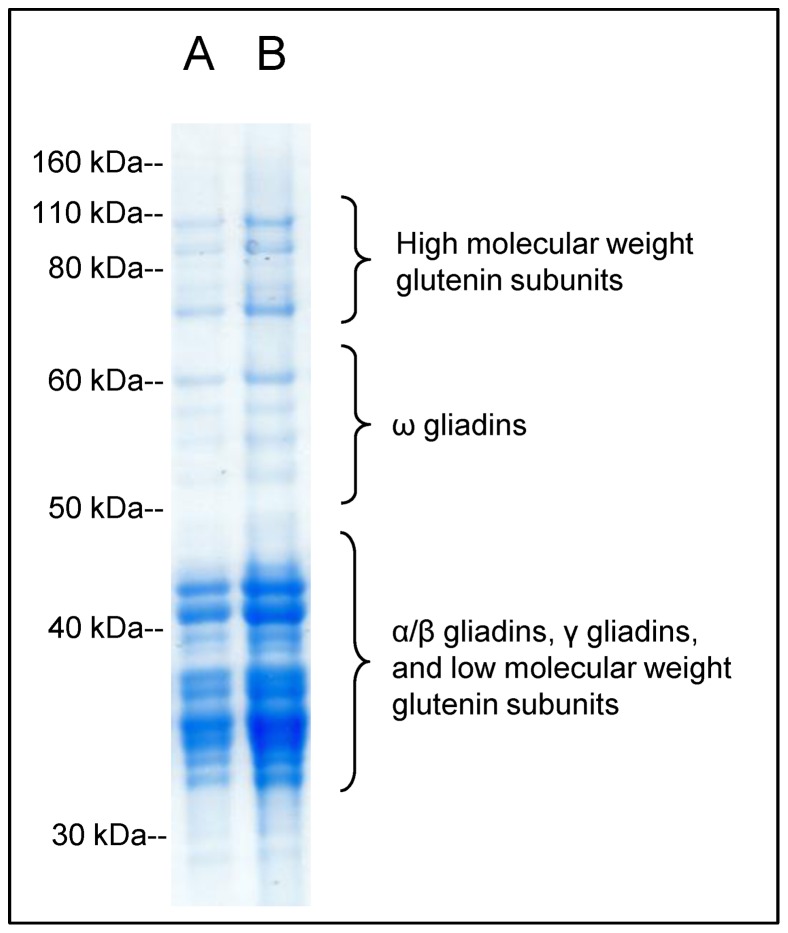
Gel electrophoresis profile of the PWG gliadin preparation used for the anti-gliadin antibody assays. A) 5 µg of protein loaded; B) 10 µg of protein loaded.

The gel electrophoresis profile for the PWG gliadin used in anti-gliadin antibody assays indicated the presence of all main types of gliadin proteins, α/β, γ, and ω. The mixture also contained high and low molecular weight glutenin subunits (Fig. 1).

### Antibody Levels

**Figure 2:**
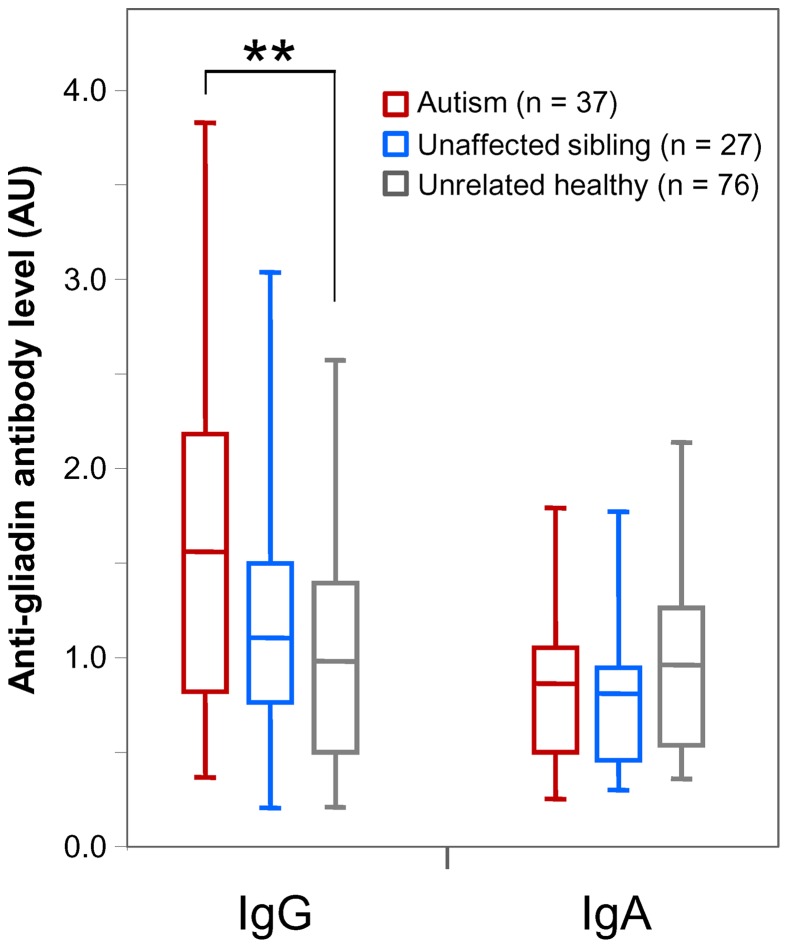
Comparison of levels of IgG and IgA antibody to gliadin in children with autism, their unaffected siblings, and unrelated healthy controls. Boxed segments represent the middle 50% of the data. Whiskers indicate the range of data. Large horizontal bars indicate mean value of the data. ** = *p*<0.01.

Mean levels of IgG and IgA class antibodies to gliadin in patient and control groups are presented in Fig. 2. Children with autism exhibited significantly elevated levels of IgG antibody to gliadin when compared with unrelated healthy controls or when compared with the combination of unaffected siblings and unrelated healthy controls (*p*<0.01). The difference remained significant after adjusting for the covariates of age, gender, and race (*p*<0.01). The anti-gliadin IgG differences between the children with autism and their unaffected siblings, and between the siblings and unrelated healthy controls, did not reach statistical significance. Based on the stated cutoff for positivity (95th percentile of the healthy control group), 8/33 (24.2%) of the children with autism, excluding those who reported being on gluten-free diet, 8/37 (21.6%) of all autistic children, including those on gluten-free diet, 2/27 (7.4%) of unaffected siblings, and 4/76 (5.3%) of unrelated healthy children were positive for IgG anti-gliadin antibody, indicating a significantly higher frequency in those with autism compared to unrelated healthy controls (*p*<0.01). Children with autism had increased odds of having elevated IgG antibody to gliadin in comparison to healthy controls (odds ratio: 4.97; 95% confidence interval: 1.39–17.8). The differences in levels of IgA antibody to gliadin among the three groups were not significant.

All patients and controls were also tested for the currently recommended full panel of the most sensitive and specific serologic markers of celiac disease, including IgA antibody to TG2, IgG antibody to deamidated gliadin, and IgA antibody to deamidated gliadin. None of the individuals in any group were positive for IgA antibody to TG2. Two of 37 autistic children, 3 of 27 unaffected siblings, and none of 76 unrelated healthy controls had values above the manufacturer’s assigned cutoff for IgG antibody to deamidated gliadin. Similarly, none of 37 autistic children, 1 of 27 unaffected siblings, and 1 of 76 unrelated healthy controls were positive for IgA antibody to deamidated gliadin.

All four individuals who were on gluten-free diet were negative for anti-gliadin, anti-deamidated gliadin, and anti-TG2 antibodies.

### HLA Typing

In the group of children with autism, 18/37 (48.6%) were positive for HLA-DQ2 and/or -DQ8 (6 DQ2, 12 DQ8). There was no clear association between antibody to gliadin and the presence of celiac disease-associated HLA-DQ2/DQ8 in patients with autism: 3/8 (37.5%) of the anti-gliadin antibody-positive individuals with autism displayed HLA-DQ2 and/or DQ8 (2 DQ2, 1 DQ8), while 15/29 (51.7%) of those below the cutoff for antibody positivity had DQ2 and/or DQ8. Neither of the two patients with autism who were positive for IgG anti-deamidated gliadin antibody had DQ2 or DQ8. About 95% or more of celiac disease patients carry HLA-DQ2 and/or -DQ8, compared to an estimated 40% of the U.S. general population [26].

### GI Symptoms

**Figure 3:**
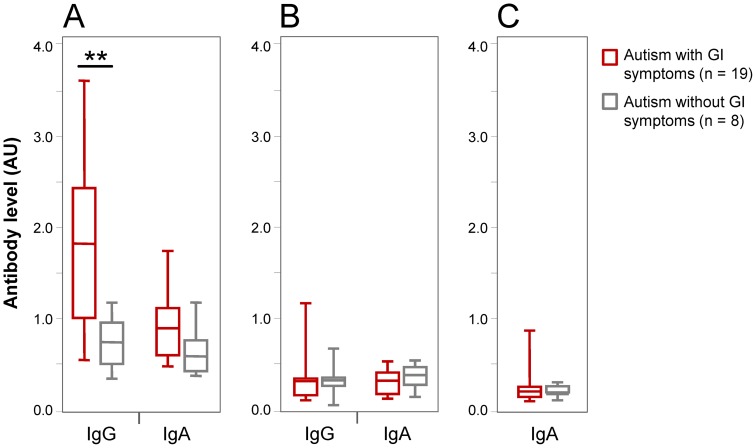
Comparison of levels of antibody to A) gliadin, B) deamidated gliadin fusion peptide, and C) human TG2 in autistic children, with and without GI symptoms. Boxed segments represent the middle 50% of the data. Whiskers indicate the range of data. Large horizontal bars indicate mean value of the data. ** = *p*<0.01.

Medical histories were available for 27 of the 37 children with autism. 19/27 (70.3%) reported persistent GI symptoms, including 10 with chronic loose stools or diarrhea, 2 with gastroesophageal reflux, 3 with frequent stools, 3 with constipation, and 1 with non-specified GI symptoms. Affected patients with GI symptoms were found to have significantly higher levels of IgG antibody to gliadin when compared to patients without GI symptoms (*p*<0.01) (Fig. 3A). This difference remained significant after adjusting for the covariates of age, gender, and race (*p*<0.01). Information on GI symptoms was available for 5 of the 8 children whose anti-gliadin antibody levels were determined to be above the cutoff. They included 3 with chronic loose stools or diarrhea, 1 with frequent stools, and 1 with constipation.

There was no significant difference in the levels of IgA antibody to gliadin (Fig. 3A), IgG and IgA antibodies to deamidated gliadin (Fig. 3B), and IgA antibody to TG2 (Fig. 3C) between patients with GI complaints and those without. One autism patient with GI symptoms was positive for IgG antibody to deamidated gliadin, while the remaining patients in both groups were negative for all other markers.

## Discussion

The aim of this study was to carry out a comprehensive analysis of markers of celiac disease and gluten sensitivity in a group of children with autism who had been diagnosed according to strict criteria and defined instruments. Our data indicate that children with autism have higher levels of IgG antibody to gliadin compared to healthy controls. In addition, among patients with autism, the antibody response to gliadin was greater in those with GI symptoms. However, in contrast to patients with celiac disease, no association was observed between the elevated anti-gliadin antibody level and the presence of highly specific serologic markers of celiac disease or HLA-DQ2/DQ8. The findings indicate that the observed anti-gliadin immune response in patients with autism is likely to involve a mechanism that is distinct from celiac disease, without the requirement for TG2 activity or antigen presentation through DQ2/DQ8 MHC molecules [27].

The data from this study should be interpreted with caution. Most importantly, the observed increased IgG antibody response to gliadin does not necessarily indicate sensitivity to gluten or any pathogenic role for antibodies to gliadin in the context of autism. In addition, the results do not rule out the possibility of moderately increased prevalence of celiac disease among children with autism, especially as duodenal biopsy, the gold standard for definitive diagnosis of celiac disease, was not performed. However, considering the excellent sensitivity and specificity of anti-TG2 and (and to a lesser extent anti-deamidated gliadin) antibodies, as well as the high negative predictive value of HLA-DQ2/DQ8 markers for celiac disease, it can be concluded with high certainty that the overwhelming majority of autism patients with elevated antibody to gliadin do not have celiac disease. If future studies prove the existence of sensitivity to gluten in a subset of patients with autism, the gluten-associated symptoms in such individuals may fall within the spectrum of “non-celiac gluten sensitivity” [28].

Compared to previous reports examining the link between celiac disease/gluten sensitivity and autism, this study is unique in several ways. First, a shortcoming in earlier studies has been the lack or incompleteness of suitable age-matched healthy control groups necessary for this type of analysis. In this work, the antibody levels in children with autism were compared to two separate pediatric control groups: unaffected siblings of the same patients, as well as a larger cohort of unrelated healthy children. Second, previous reports have used specimens from more heterogeneous groups of patients generally recruited at local hospitals or clinics, and while most report the use of DSM diagnostic criteria, it is unclear which test(s) informed the final diagnosis of autism. In contrast, the samples in this study were acquired from a well-recognized repository of biomaterials (AGRE), which is managed by the world’s largest autism advocacy organization and has been utilized in various past research projects. The associated AGRE database includes information about family pedigree, scores from various tests and questionnaires, and medical histories for many of the patients for which biospecimens are available. Patients in this study were selected only if they were identified as having autism according to two separate instruments, ADOS and ADI-R, thus greatly increasing the likelihood of accurate diagnosis.

A limitation of this study is that we could not control for geographical distribution, socioeconomic status, or diet of the research participants. These factors may contribute to levels of antibodies against dietary and other antigens in patients and controls. In addition, information on GI symptoms was available only for some patients and none of the controls. Access to such data would have strengthened the study’s finding regarding the association between GI symptoms and anti-gliadin antibody levels. As such, the conclusions of this study should be considered preliminary, requiring further confirmation in larger and better-characterized cohorts of patients and controls.

We can consider some possibilities to explain the higher anti-gliadin antibody levels found in the cohort of children with autism. Previously, associations between autism and increased GI symptoms, as well as impaired intestinal permeability, have been reported [9], [10], [29]. Increased intestinal permeability resulting from damage to the intestinal epithelial barrier in those with autism may be responsible for increased exposure of the immune system to partially digested gluten fragments, resulting in the detected increase in antibody response. The observation here that anti-gliadin antibody reactivity is elevated in patients with GI symptoms lends some support for this idea. At the same time, the fact that the higher anti-gliadin antibodies in autistic children were limited to the IgG isotype, without a concomitant rise in IgA, may imply a non-mucosal and/or gluten-independent origin for the observed antibody reactivity. One possibility is that the IgG-specific antibody response in children with autism would have been triggered by ingested gluten at some point in the past, but no longer dependent on continuous mucosal exposure to the proteins. Alternatively, the detected anti-gliadin antibodies may be unrelated to gluten as the immunogen. Various immune abnormalities have been demonstrated in autistic children, including increased antibody reactivity to autoantigens [30]–[32]. It is conceivable that certain autism-associated autoantibodies, the exact targets of which are yet to be identified, would cross-react with one or more gluten proteins and contribute to the detected difference in anti-gliadin antibody level between patients and controls. Circulation levels of such antigen-independent or gluten cross-reactive antibodies would not be expected to respond to dietary gluten restriction.

Results of this study are intriguing in the context of disease pathophysiology and biomarker identification. The observed increase in antibody reactivity to gliadin in over one fifth of the autism cohort points to potential shared genetic and/or environmental associations in a sizable subset of patients. As such, the generated data provide an impetus to further examine the affected patient subset for additional immunologic and genomic clues. It is possible that, in a subset of children with autism, the condition is associated with antibody reactivity to a unique set of gluten proteins that would be significantly different from the pattern of anti-gliadin antibody response in celiac disease and other conditions. This specific pattern of antibody reactivity may be useful as a source of biomarkers. A unique antibody response to particular gluten molecules could also be associated with specific HLA genes in that disease subset.

In conclusion, the increased anti-gliadin antibody response in autism and its association with GI symptoms points to a potential mechanism involving immunologic and/or intestinal permeability abnormalities in a subset of patients. The observed antibody reactivity to gliadin in most children with autism appears to be unrelated to celiac disease. Therefore, the heightened immune response to gluten in autism deserves further attention and research in determining its utility as a source of biomarkers and clues regarding disease pathophysiology. Better understanding of this immune response may offer novel markers for the identification of subsets of patients who would be responsive to specific treatment strategies.
